# Maternal diabetes-associated neural tube defects: evidence for β-hydroxybutyrate-related HDAC1/3-H3K27ac-MEST-LRP6 dysregulation

**DOI:** 10.3389/fphys.2026.1881715

**Published:** 2026-09-03

**Authors:** Wenjia Ma, Yufei Wang, Ziyu Li, Zizhen Liu, Aili Fang

**Affiliations:** 1 Anesthesiology College of Shanxi Medical University, Taiyuan, Shanxi, China; 2 Shanxi Bethune Hospital, Shanxi Academy of Medical Sciences, Third Hospital of Shanxi Medical University, Tongji Shanxi Hospital, Taiyuan, China; 3 Department of Anesthesiology, The First Hospital of Shanxi Medical University, Taiyuan, Shanxi, China; 4 Biochemistry and Molecular Biology Laboratory, Shanxi Medical University, Taiyuan, Shanxi, China

**Keywords:** diabetes, H3K27ac, MEST, neural tube defects, β-hydroxybutyrate

## Abstract

Maternal diabetes is an important risk factor for embryonic developmental defects, including neural tube defects (NTDs). Elevated β-hydroxybutyrate (BHB), a major ketone body increased under diabetic metabolic conditions, has been implicated in abnormal embryonic development, but its relationship with epigenetic regulation during neurulation remains incompletely defined. In this study, maternal diabetes was induced in pregnant ICR mice by intraperitoneal streptozotocin (STZ; 200 mg/kg) at embryonic day 5.5. In the final *in vivo* model, 18 control dams produced 216 embryos, whereas 20 diabetic dams produced 250 embryos, including 43 NTD-affected embryos (17.2%). Diabetic pregnancies showed elevated maternal serum BHB, and BHB levels were also increased in brain tissue from diabetic NTD embryos. Ex vivo whole-embryo culture of E8.5 embryos in rat serum supplemented with BHB (2 or 4 mM) showed impaired embryonic growth and NTD-like morphological abnormalities at 4 mM BHB, together with increased embryonic brain BHB content. At the molecular level, diabetic NTD brain tissue and BHB-treated HT-22 neuronal cells showed reduced HDAC1/3 expression and increased H3K27ac. MEST expression was increased whereas mature LRP6 was decreased. HDAC1 or HDAC3 overexpression in BHB-treated HT-22 cells reduced H3K27ac and partially reversed MEST and mature LRP6 changes. These findings support an association between elevated BHB and HDAC1/3-H3K27ac-MEST-LRP6 dysregulation in maternal diabetes-associated NTDs. Because direct chromatin-occupancy and MEST functional manipulation experiments were not performed, the proposed pathway should be interpreted as a potential mechanism requiring further validation.

## Introduction

Neural tube defects (NTDs) are severe congenital malformations caused by the developmental failure of neural tube closure during embryonic development (Palomaki et al., 2020). The neural tube formation is a critical early event in nervous system development. In mice, neural tube closure occurs over two days: the anterior neuropore closes at E8.5–E9.5, followed by the posterior neuropore at E9.5–E10.5. NTDs significantly impact population health and quality of life, underscoring the importance of elucidating their pathogenesis.

Early research on NTDs focused on identifying susceptibility genes. Approximately 400 NTD-associated genes have been reported in animal models (Chen et al., 2018). However, due to interspecies differences, the relevance of these genes in human NTDs remains unclear. Moreover, known genetic mutations account for only a small fraction of NTD cases (Lynch, 2005; Harris and Juriloff, 2010). Current evidence suggests that NTDs may arise from genetic predisposition and environmental factors, particularly the maternal nutrition and medication exposure (Zhang Q. et al., 2018). Maternal metabolic disturbances are a major environmental contributor to NTDs. One of the classic examples is folate deficiency, which leads to DNA hypomethylation and reduced histone methylation, increasing NTD risk (Chang et al., 2021). Other metabolic defects, such as elevated homocysteine and inositol deficiency, would also result in severe NTD via histone homocysteinylation and impaired phosphatidylinositol metabolism (Copp et al., 2013; Zhang Q. et al., 2018).

Diabetes is a complex metabolic disorder characterized by systemic dysregulation and multi-organ complications (Haines et al., 2018; Sowton et al., 2019; Zhao et al., 2012). Offspring of diabetic mothers exhibit a higher incidence of birth defects than offspring of non-diabetic mothers (Correa et al., 2008; Ornoy et al., 2015). Multiple mechanisms have been implicated in diabetic embryopathy, including oxidative stress, mitochondrial dysfunction, endoplasmic reticulum stress, apoptosis, autophagy dysregulation, impaired Wnt signaling, and epigenetic remodeling (Cao et al., 2016; Pacini et al., 2004; Prakasam et al., 2014; Gu et al., 2016; Wang et al., 2017). These pathways are not mutually exclusive. For example, oxidative stress can aggravate endoplasmic reticulum stress and apoptosis, while metabolic intermediates can influence chromatin-modifying enzymes and histone marks. Therefore, a balanced interpretation of diabetes-associated NTDs should consider both established stress-response pathways and metabolic-epigenetic mechanisms.

Ketone bodies, including β-hydroxybutyrate (BHB), acetoacetate, and acetone, serve as alternative energy substrates during glucose scarcity (Dabek et al., 2020). BHB is the predominant circulating ketone body and has been implicated in embryonic malformations in previous whole-embryo culture studies (Hunter et al., 1987). In addition to its metabolic role, BHB can act as an endogenous inhibitor of class I histone deacetylases (HDACs), thereby influencing histone acetylation and transcriptional regulation (Shimazu et al., 2013; Li et al., 2020; Shirian et al., 2024). H3K27ac is enriched at active promoters and enhancers and is commonly associated with transcriptional activation (Gates et al., 2017; Ding et al., 2017). Because neural tube closure depends on precise spatial and temporal control of neurodevelopmental gene expression, abnormal H3K27ac regulation may contribute to NTD susceptibility. Previous studies have also linked altered histone acetylation to diabetic embryopathy and NTD-associated gene-expression changes (Shimazu et al., 2013; Gu et al., 2016; Gates et al., 2017). Collectively, these findings provided a strong biological rationale for focusing on BHB as a candidate metabolic mediator linking the diabetic metabolic environment to epigenetic dysregulation during embryonic development. Accordingly, BHB was investigated in the present study within a hypothesis-driven framework based on prior metabolic, embryological, and epigenetic evidence, rather than being identified through an unbiased metabolomic discovery approach (Hunter et al., 1987; He et al., 2023).

Beyond its metabolic role, BHB modulates neuroprotection, oxidative stress, and epigenetic regulation (Shirian et al., 2024; Chen et al., 2017). Recent work identifies BHB as an endogenous class I histone deacetylase (HDAC) inhibitor (Shimazu et al., 2013). Class I HDACs (HDAC1, 2, 3, 8) regulate histone acetylation dynamics, with HDAC1/3 specifically influencing H3K27ac levels—a marker of transcriptional activation enriched at promoters/enhancers. Elevated H3K27ac has been linked to NTDs (Shimazu et al., 2013; Gates et al., 2017).

Based on these observations, we hypothesized that maternal diabetes-associated BHB elevation may contribute to NTD development by altering HDAC1/3 expression and H3K27ac-associated gene regulation in embryonic neural tissue. Bioinformatic inspection using the UCSC Genome Browser suggested H3K27ac enrichment near the Mest promoter, and previous work reported that MEST can regulate LRP6 maturation. We therefore examined whether BHB-related HDAC1/3-H3K27ac changes are associated with altered MEST expression and mature LRP6 levels in diabetic NTD embryos and in a BHB-treated neuronal cell system.

In this study, we established an STZ-induced maternal diabetes model and assessed embryonic morphology, maternal and embryonic BHB levels, HDAC1/3 expression, H3K27ac, MEST expression, and mature LRP6. We also used ex vivo whole-embryo culture to test the developmental effect of BHB exposure and HT-22 cells to evaluate a limited mechanistic rescue by HDAC1/3 overexpression. The data support a potential BHB-associated epigenetic mechanism in diabetes-associated NTDs, while also identifying steps that require direct validation in future studies.

## Materials and methods

### Animal

Clean-grade ICR mice aged 7–10 weeks were maintained under standard laboratory conditions with controlled temperature, humidity, a 12-h light/dark cycle, and free access to food and water. After one week of acclimatization, estrous female mice were paired with male mice at 5:00 PM, and vaginal plugs were checked the following morning. Noon on the day a plug was detected was designated embryonic day 0.5 (E0.5). All animal procedures were approved by the ethics committee (SBQDL-2022-057).

To induce maternal diabetes during neurulation while avoiding effects on implantation, pregnant mice received a single intraperitoneal injection of STZ at E5.5. STZ was prepared freshly on ice and protected from light. The final dose used for the main experiment was 200 mg/kg. This dose was selected based on preliminary dose-optimization experiments in E5.5 pregnant ICR mice: 180, 190, 200, 210, and 220 mg/kg were compared, and 200 mg/kg produced a high diabetic modeling rate (13/15 pregnant mice, 86.6%) with fewer resorptions than higher doses and a robust NTD phenotype. Vehicle-injected pregnant mice served as controls.

Blood glucose was measured from tail blood beginning three days after STZ injection. Pregnant mice with blood glucose levels consistently exceeding 16.7 mM for three consecutive days were classified as diabetic. Body weight was recorded before injection and during model monitoring.

At E10.5, pregnant mice were euthanized and embryos were dissected in pre-chilled PBS. Embryo number, resorption number, embryo size, malformation site, and NTD incidence were recorded. In the final model, the control group included 18 pregnant mice and 216 embryos, with 1 NTD embryo (0.46%); the diabetic group included 20 pregnant mice and 250 embryos, with 43 NTD embryos (17.2%) and 10 resorptions. Embryos were photographed under standardized conditions and then stored in liquid nitrogen or fixed in 4% paraformaldehyde according to downstream analyses.

### *In vitro* rat embryonic culture

For rat serum preparation, male Sprague-Dawley rats weighing approximately 500 g were anesthetized with isoflurane, and blood was collected by cardiac puncture into serum separator tubes. Serum was separated after clotting and stored at -80 °C until use.

For ex vivo whole-embryo culture, intact E8.5 ICR mouse embryos with ectoplacental cones were dissected under sterile conditions and randomly allocated to treatment groups. Heat-inactivated rat serum was used as the culture medium. The serum was divided into three groups: 0 mM BHB (control), 2 mM BHB, and 4 mM BHB. Treatment media were filtered through a 0.22 μm sterile filter and added to sterile rotating culture bottles. Embryos were cultured at 37 °C and 30 rpm. Bottles were initially flushed with 5% O2 gas, and gas exchange was performed every 4 h. At E9.5, media were replaced with fresh treatment-matched serum, and the gas mixture was switched to 20% O2 until E10.5. Thus, embryos were exposed to BHB for 48 h, with treatment renewed once at E9.5.

### ELISA

BHB concentrations in maternal serum and embryonic brain homogenates were measured using a commercial β-hydroxybutyrate assay kit (Shanghai Xitang Co., Ltd., Shanghai, China) according to the manufacturer’s instructions. Standards were prepared at 4, 2, 1, 0.5, 0.25, 0.125, 0.063, and 0 mM. For each well, 5 μL of standard or sample was added to the 96-well plate, substrate working solution was added, and absorbance was read before and after enzyme-conjugate incubation. Net absorbance was calculated as A2 minus A1, and sample BHB concentrations were calculated from the standard curve.

### Cell culture

HT-22 cells were used as a neuronal cell system for controlled BHB exposure and HDAC1/3 overexpression experiments. Cells were cultured in DMEM supplemented with fetal bovine serum and penicillin/streptomycin; no additional mitogenic factors were used. For BHB treatment, cells were exposed to complete medium containing 0, 2, or 4 mM BHB for 24 h. For rescue experiments, cells were seeded in 6-well plates at 1.5 x 10^5 cells per well. HDAC1 or HDAC3 overexpression plasmids were transfected using Lipofectamine 2000, with 4 μg plasmid DNA per well, and cells were analyzed 48 h after transfection. HT-22 cells are not a model of neurulation or neural tube closure; they were used only to test molecular responses to BHB and HDAC1/3 restoration in a controlled neuronal context.

### Real-time PCR

Total RNA was extracted from HT-22 cells or embryonic brain tissue using TRIzol reagent. For embryonic brain tissue, 2–3 embryonic brain samples were pooled per extraction tube when needed. RNA was treated with gDNA wiper mix and reverse-transcribed using qRT SuperMix (50 °C for 15 min and 85 °C for 2 min). Quantitative PCR was performed with an initial denaturation at 95 °C for 30 s, followed by 40 amplification cycles of 95 °C for 10 s and 60 °C for 30 s, with melt-curve analysis performed after amplification. β-actin was used as the reference gene, and relative mRNA expression was calculated using the 2^-ΔΔCt method. Primer sequences are listed in **Table 1**.

**Table 1:** Primer sequences used for quantitative real-time PCR.

Gene	Primer
*Hdac1*	F: 5’-AGTCTGTTACTACTACGACGGG-3’ R: 5’-TGAGCAGCAAATTGTGAGTCAT-3’
*Hdac3*	F: 5’-GGCCATTAGTGAGGAACTTCC-3’ R: 5’-TCCACATCACTTTCCTTGTCG-3’
*Mest*	F: 5’-CCATGGTGCGCCGAGATCGCTTGC-3’ R: 5’-CCAGCTCAGAAGGAGTTGATGAAGCCC-3’
*β-actin*	F: 5’- GTGACGTTGACATCCGTAAAGA -3’ R: 5’- GCCGGACTCATCGTACTCC-3’

### Western blot

Protein was extracted from HT-22 cells or embryonic brain tissue using RIPA lysis buffer supplemented with protease inhibitor. For embryonic tissue, lysis buffer was added at approximately 8 μL per 1 mg tissue. Samples were lysed at 4 °C, sonicated, and centrifuged at 13,000 rpm for 20 min. Protein concentration was determined using the BCA method. Equal amounts of protein were separated by SDS-PAGE and transferred to PVDF membranes. Membranes were incubated with primary antibodies against HDAC1, HDAC3, H3K27ac, MEST, LRP6, and the loading control overnight at 4 °C, followed by HRP-conjugated secondary antibody incubation at room temperature for 2 h. Bands were visualized using ECL and quantified using ImageJ as the ratio of target-band intensity to loading-control intensity.

### Immunofluorescence

For immunofluorescence, HT-22 cells were seeded in 24-well plates, treated as indicated, washed with PBS, fixed with 4% paraformaldehyde for 30 min, permeabilized with 0.1% Triton X-100, and blocked with 10% goat serum for 30 min. Cells were incubated with primary antibody overnight at 4 °C and then with Goat anti-Rabbit IgG-Cy3 secondary antibody (Absin, Shanghai, China) at 37 °C for 1 h, followed by DAPI staining for 5 min. Images were acquired under identical exposure settings within each experiment, and fluorescence intensity was quantified using ImageJ.

### Immunohistochemistry

For immunohistochemistry, normal embryos and diabetic NTD embryos were fixed in 4% paraformaldehyde for 24 h, dehydrated, embedded in paraffin, and serially sectioned. Sections containing comparable neural tube/brain regions were selected, deparaffinized, rehydrated, subjected to antigen retrieval by microwave heating for 10 min, blocked, and incubated with H3K27ac primary antibody overnight. Sections were then incubated with a rabbit/mouse universal secondary antibody for immunohistochemistry (Zhongshan Golden Bridge, Beijing, China) at 37 °C for 30 min. DAB staining was monitored under the microscope, and sections were counterstained with hematoxylin. H3K27ac staining intensity was quantified using ImageJ under identical image-acquisition settings.

### Statistic

Statistical analysis was performed using SPSS software (version 16.0). Data are presented as mean ± standard deviation (mean ± SD). Normality and homogeneity of variance were assessed before parametric testing. Student’s t-test was used for two-group comparisons, and one-way or two-way ANOVA was used for comparisons involving more than two groups or two factors, followed by appropriate *post hoc* tests when assumptions were met. Non-parametric tests were used when data did not meet parametric assumptions. A P-value < 0.05 was considered statistically significant.

## Result

### Maternal diabetes induced the BHB-mediated brain development defects

**Figure 1:**
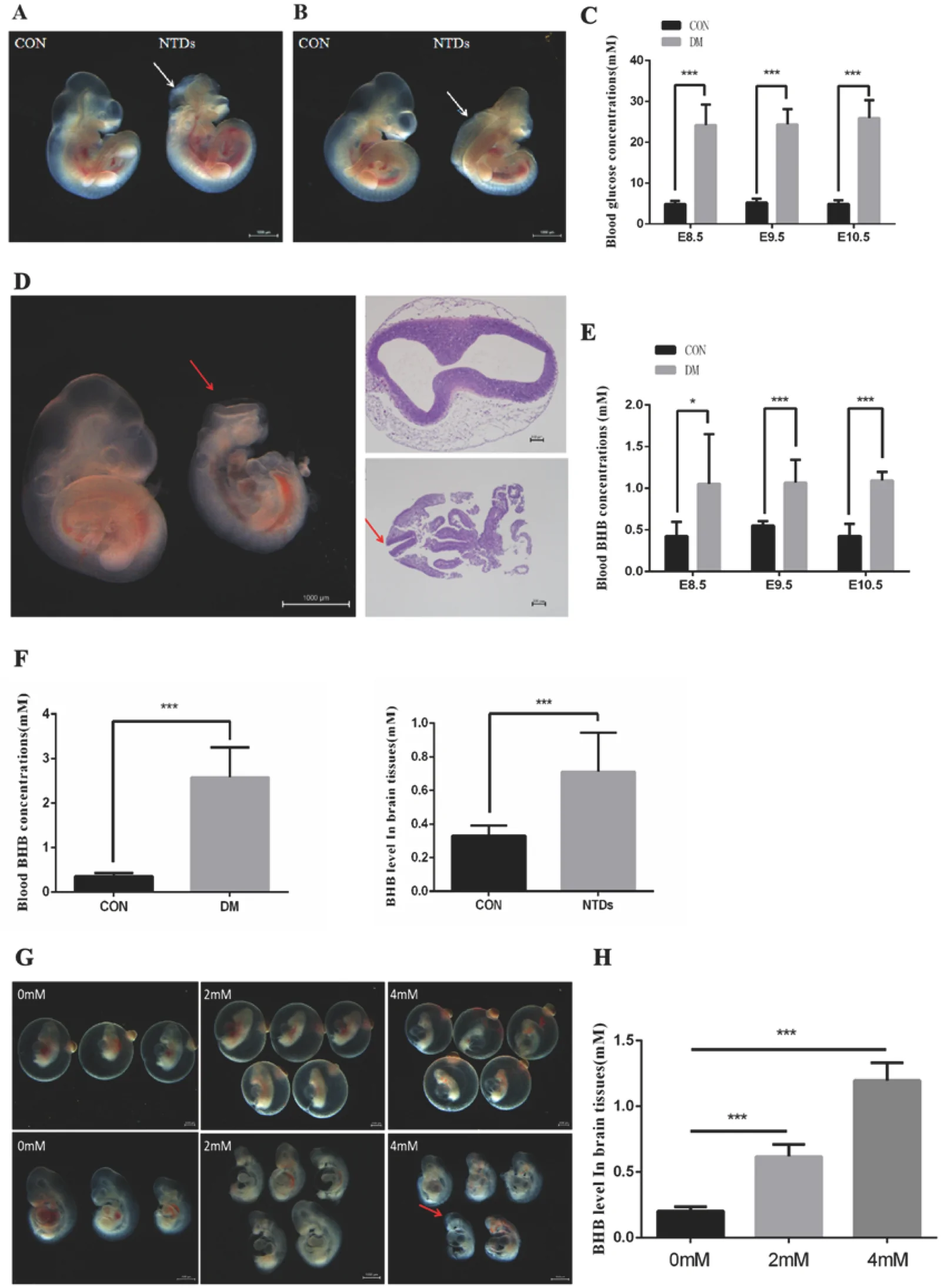
Maternal diabetes and BHB exposure are associated with embryonic brain developmental defects. **(A, B)** Representative bright-field images showing NTD phenotypes, including exencephaly **(A)** and spina bifida **(B)**, in embryos from diabetic pregnancies. **(C)** Maternal blood glucose levels after STZ modeling at E8.5, E9.5, and E10.5; n = 8 dams per group; data are mean ± SD; ***P < 0.001. **(D)** Representative bright-field and H&E images of control embryos and diabetic NTD embryos. **(E)** Maternal serum BHB levels at E8.5, E9.5, and E10.5 measured by ELISA; n = 8 dams per group; data are mean ± SD; *P < 0.05, ***P < 0.001. **(F)** E13.5 maternal serum BHB and embryonic brain BHB levels in control and diabetic/NTD groups; n = 8 per group; data are mean ± SD; ***P < 0.001. **(G)** Representative images of E8.5 embryos cultured ex vivo for 48 h in rat serum containing 0, 2, or 4 mM BHB. **(H)** BHB levels in embryonic brain tissue after ex vivo BHB exposure; n = 8 per group; data are mean ± SD; ***P < 0.001.

Pregnant mice were first mated and then injected with STZ at E5.5 to induce hyperglycemia during neural tube development. This timing was chosen to avoid potential effects of diabetes on implantation while maintaining hyperglycemia during neurulation. In the final experiment, 18 control dams produced 216 embryos, whereas 20 diabetic dams produced 250 embryos. NTDs were observed in 43 embryos from diabetic dams (17.2%) and in 1 embryo from control dams (0.46%). Diabetic embryos showed delayed development and failed neural tube closure, including exencephaly and spina bifida (**Figures 1A, B**). Maternal blood glucose was significantly elevated during E8.5-E10.5 (**Figure 1C**). H&E staining showed complete neural tube closure in control embryos and open neural tube morphology in diabetic NTD embryos (**Figure 1D**).

Maternal serum BHB concentrations were higher in diabetic dams than in controls at E8.5, E9.5, and E10.5 (**Figure 1E**). At E13.5, both maternal serum BHB and embryonic brain BHB were increased in diabetic pregnancies/NTD embryos (**Figure 1F**).

To test the developmental effect of BHB exposure, intact E8.5 embryos were cultured ex vivo in rat serum containing 0, 2, or 4 mM BHB for 48 h. Embryos cultured in control serum developed with rich yolk sac circulation. BHB-treated embryos showed reduced growth and poorer yolk sac circulation, and NTD-like brain developmental abnormalities were observed in the 4 mM BHB group (**Figure 1G**). Embryonic brain BHB content increased after exposure to BHB-containing serum (**Figure 1H**). These data indicate that elevated BHB exposure is associated with impaired embryonic development in this ex vivo system.

### Maternal diabetes NTD suppressed HDAC1/3 signaling

**Figure 2:**
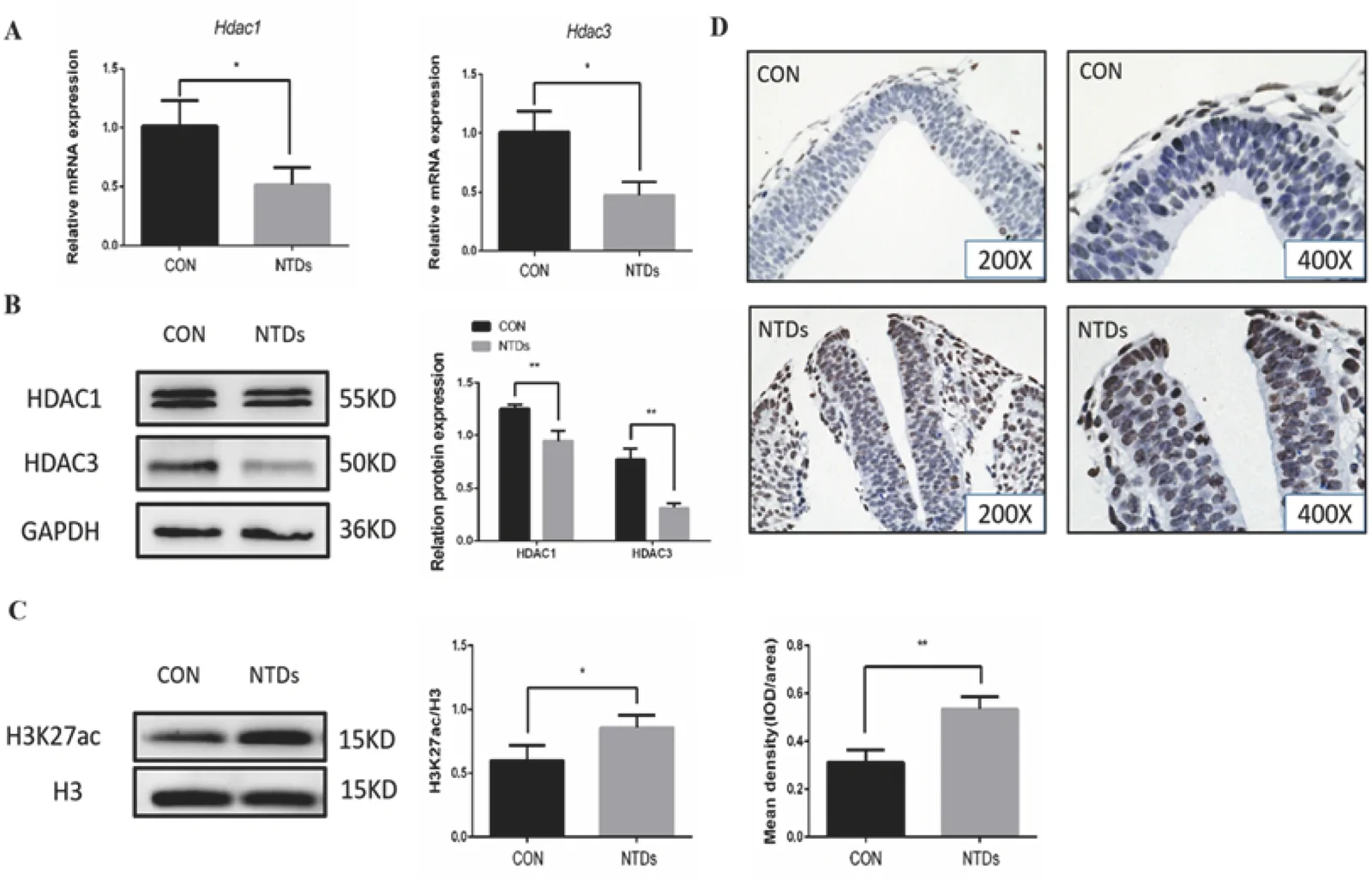
Diabetic NTD embryonic brain tissue shows reduced HDAC1/3 expression and increased H3K27ac. **(A)** RT-qPCR analysis of Hdac1 and Hdac3 mRNA expression in brain tissues from control embryos and diabetic NTD embryos; β-actin was used for normalization; data are mean ± SD; *P < 0.05. **(B)** Western blot analysis and quantification of HDAC1 and HDAC3 protein expression; signals were normalized to the loading control; **P < 0.01. **(C)** Western blot analysis and quantification of H3K27ac; *P < 0.05. **(D)** Representative H3K27ac immunohistochemistry images in control and diabetic NTD embryonic brain tissue. **(E)** Quantification of H3K27ac immunohistochemistry intensity using ImageJ under identical acquisition settings; **P < 0.01.

To determine whether diabetic NTD embryos showed altered HDAC1/3-H3K27ac signaling, we measured HDAC1, HDAC3, and H3K27ac in embryonic brain tissue. Hdac1 and Hdac3 mRNA levels and HDAC1/3 protein levels were decreased in diabetic NTD embryos compared with controls (**Figures 2A, B**). H3K27ac levels were increased by western blotting and immunohistochemistry (**Figures 2C–E**). These data demonstrate an association between diabetic NTD status, reduced HDAC1/3 expression, and elevated H3K27ac, but they do not by themselves establish developmental causality.

### BHB enhanced the expression of H3K27ac in hippocampal neurons

**Figure 3:**
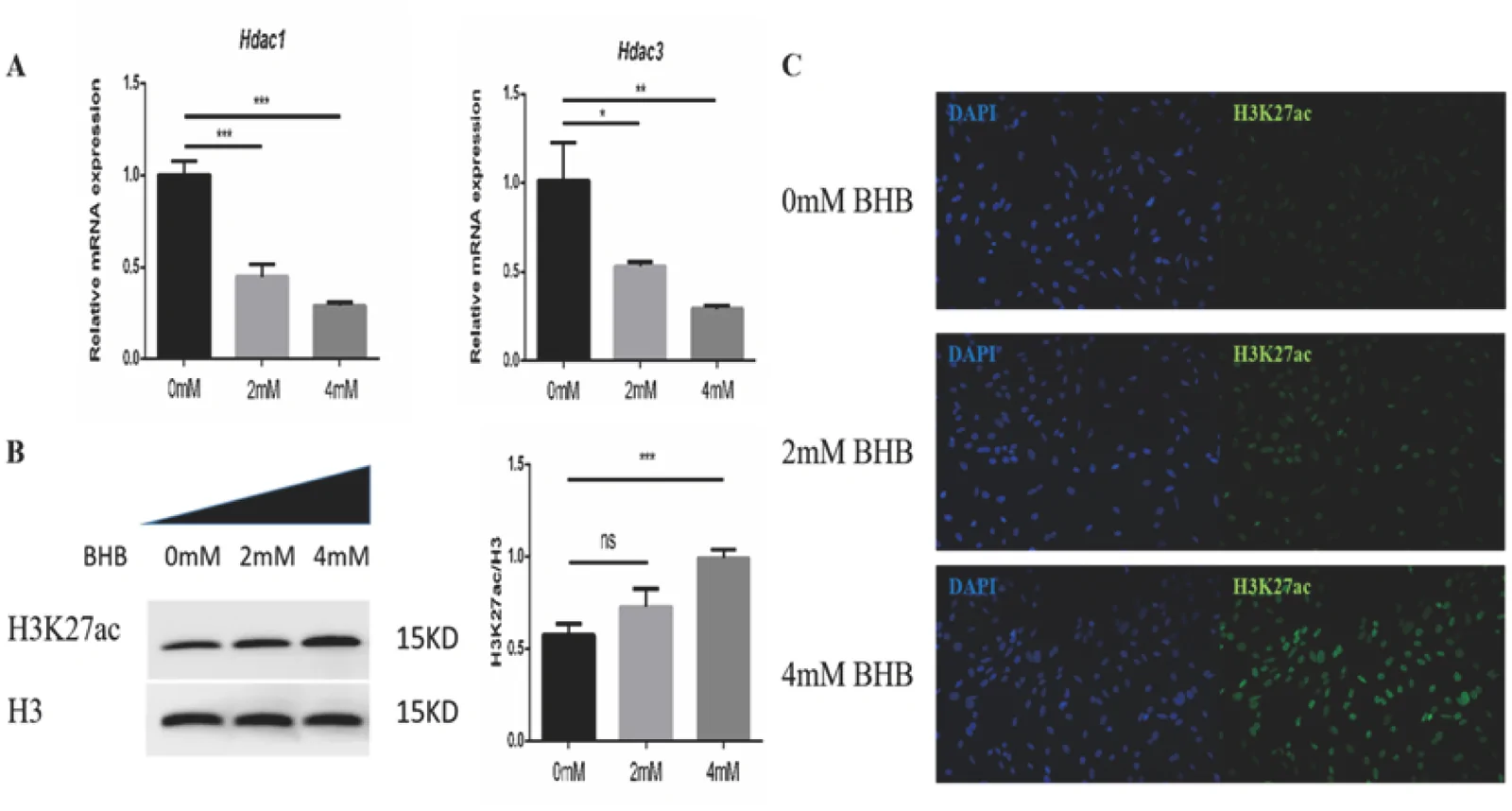
BHB treatment is associated with reduced Hdac1/Hdac3 expression and increased H3K27ac in HT-22 cells. **(A)** RT-qPCR analysis of Hdac1 and Hdac3 expression after 24 h treatment with 0, 2, or 4 mM BHB; β-actin was used for normalization; data are mean ± SD; *P < 0.05, **P < 0.01, ***P < 0.001 versus control or as indicated. **(B)** Western blot analysis and quantification of H3K27ac after BHB treatment; ***P < 0.001. **(C)** Representative immunofluorescence images of H3K27ac after BHB treatment; fluorescence intensity was quantified using ImageJ under identical acquisition settings.

HT-22 cells were used as a limited neuronal cell system to evaluate molecular responses to BHB. Because HT-22 cells are immortalized hippocampal neuronal cells rather than neuroepithelial cells undergoing neural tube closure, we do not describe them as an NTD model. Treatment with 2 or 4 mM BHB for 24 h reduced Hdac1 and Hdac3 mRNA expression, and 4 mM BHB increased H3K27ac protein and immunofluorescence intensity (**Figures 3A–C**). These results support a BHB-associated HDAC1/3-H3K27ac response *in vitro*, while the relevance to embryonic neurulation requires validation in more developmentally appropriate systems.

### Maternal diabetes NTD increased the embryonic MEST

**Figure 4:**
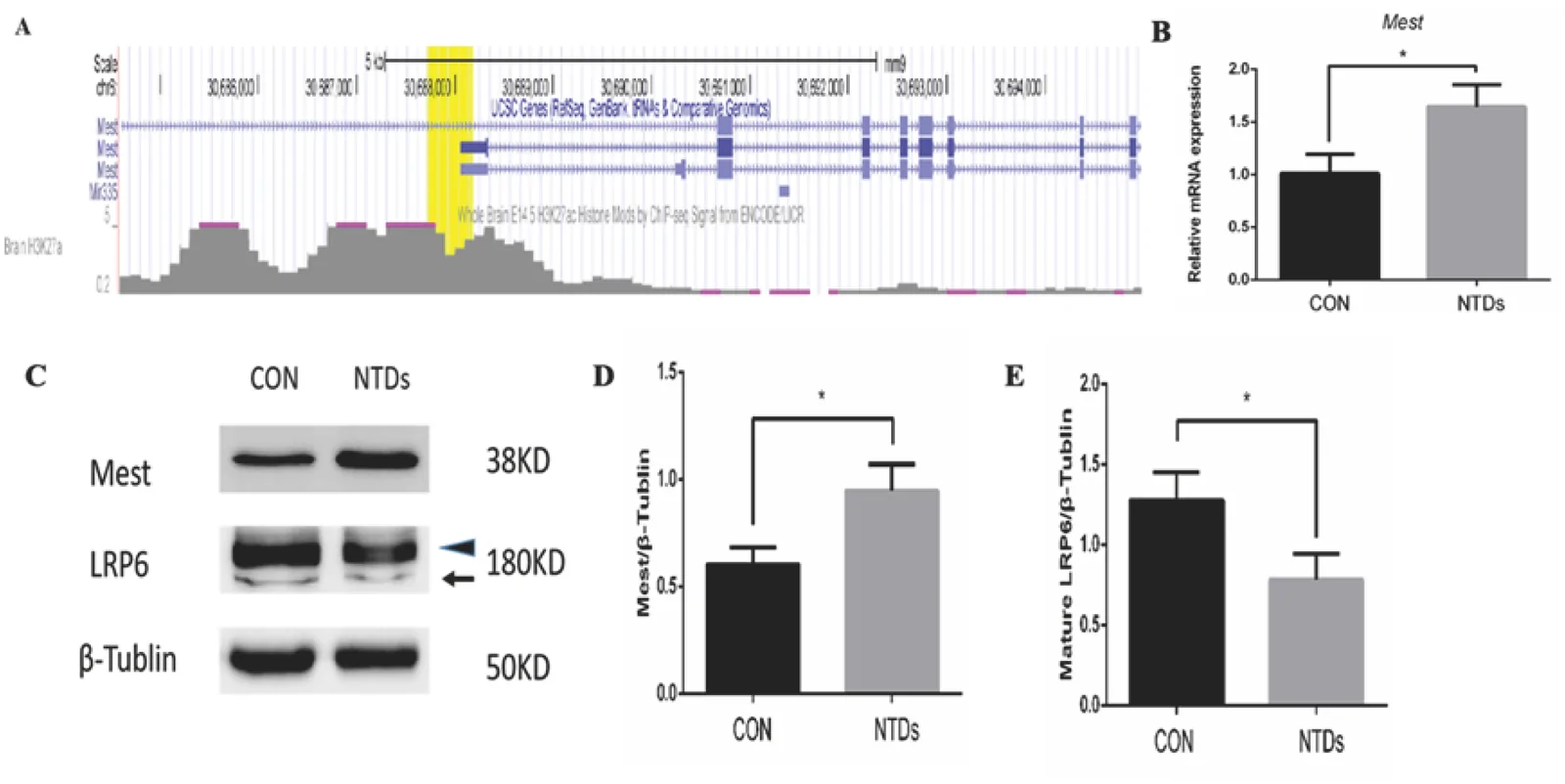
Diabetic NTD embryonic brain tissue shows increased MEST and reduced mature LRP6. **(A)** UCSC Genome Browser-based prediction showing H3K27ac enrichment near the Mest promoter; the yellow region indicates the predicted promoter region. This panel represents bioinformatic prediction, not direct experimental validation of H3K27ac occupancy. **(B)** RT-qPCR analysis of Mest expression in control and diabetic NTD embryonic brain tissue; β-actin was used for normalization; data are mean ± SD; *P < 0.05. **(C–E)**. Representative western blot bands and quantification of MEST and mature LRP6 expression in control and diabetic NTD embryonic brain tissue; signals were normalized to the loading control; *P < 0.05. The triangle indicates mature LRP6 and the arrow indicates immature LRP6.

UCSC Genome Browser inspection suggested H3K27ac enrichment near the Mest promoter (**Figure 4A**). This analysis is a bioinformatic prediction rather than direct experimental evidence of H3K27ac occupancy. Mest mRNA and MEST protein were increased in diabetic NTD embryonic brain tissue, whereas mature LRP6 protein was decreased (**Figures 4B–E**). These findings are consistent with a potential relationship among H3K27ac, MEST expression, and LRP6 maturation, but direct H3K27ac promoter-occupancy assays and MEST functional manipulation are required to establish causality.

### BHB triggered H3K27ac-mediated MEST expression

**Figure 5:**
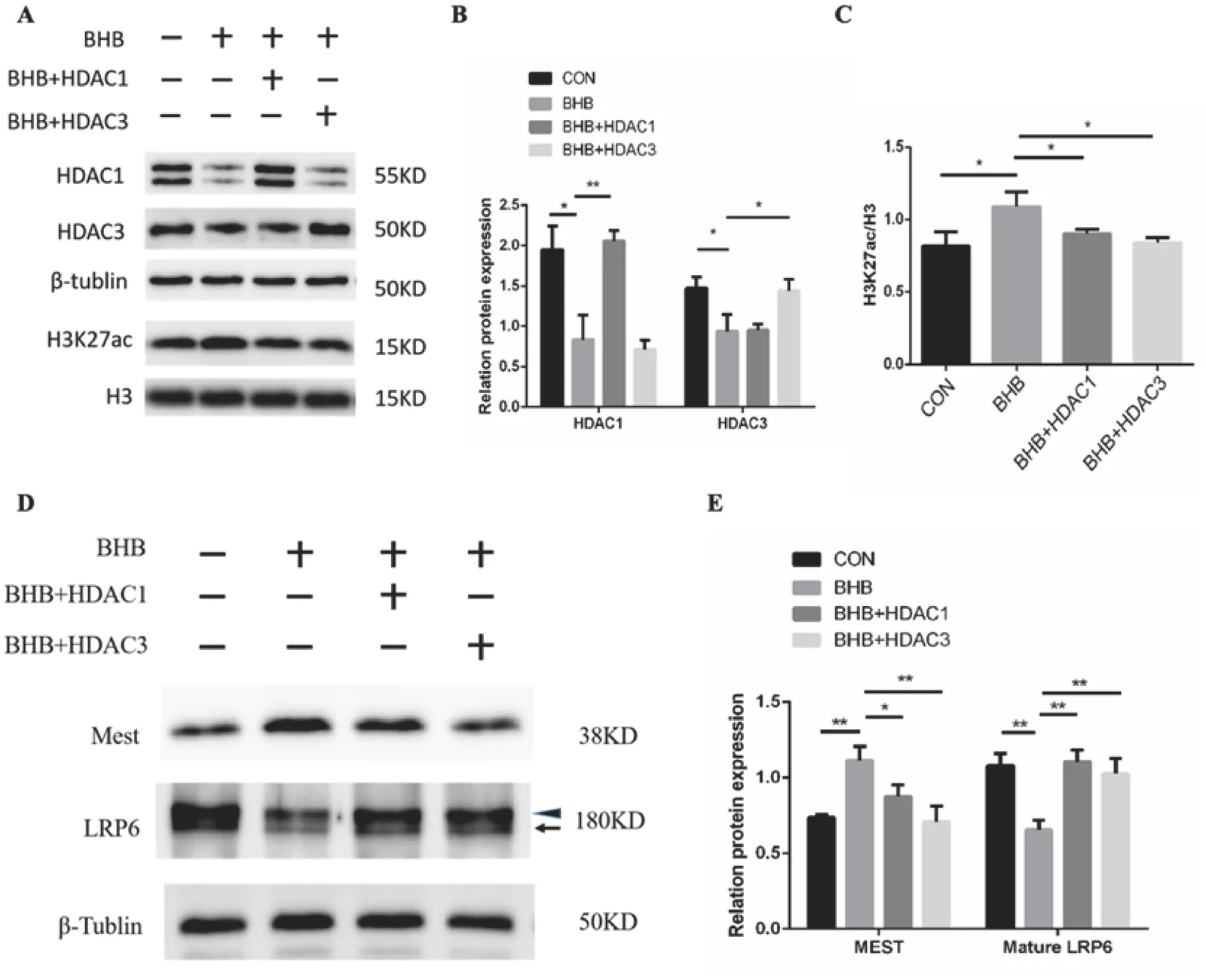
HDAC1 or HDAC3 overexpression partially reverses BHB-associated H3K27ac, MEST, and mature LRP6 changes in HT-22 cells. **(A–C)**. Representative western blot bands and quantification of HDAC1, HDAC3, and H3K27ac after HDAC1 or HDAC3 overexpression in BHB-treated cells. Cells were transfected with HDAC1 or HDAC3 overexpression plasmids and analyzed 48 h after transfection; *P < 0.05, **P < 0.01. **(D, E)**. Representative western blot bands and quantification of MEST and mature LRP6 protein levels in the same experimental groups; *P < 0.05, **P < 0.01.

To test whether restoring HDAC1/3 could attenuate BHB-associated molecular changes, HT-22 cells treated with BHB were transfected with HDAC1 or HDAC3 overexpression plasmids. HDAC1/3 overexpression reduced H3K27ac levels and partially reversed the BHB-associated increase in MEST and decrease in mature LRP6 (**Figures 5A–E**). These rescue data support a regulatory link between HDAC1/3, H3K27ac, MEST, and mature LRP6 in HT-22 cells. However, because downstream Wnt/β-catenin activity and MEST gain- or loss-of-function were not directly tested, these data should not be interpreted as definitive proof of the full pathway in neural tube closure.

## Discussion

This study examined whether elevated BHB under maternal diabetic conditions is associated with epigenetic and signaling changes relevant to NTDs. We found that diabetic pregnancies showed increased maternal and embryonic BHB, that BHB exposure impaired development in ex vivo embryo culture, and that diabetic NTD embryos and BHB-treated HT-22 cells showed reduced HDAC1/3 expression, elevated H3K27ac, increased MEST, and reduced mature LRP6. These findings support a potential BHB-associated HDAC1/3-H3K27ac-MEST-LRP6 mechanism, but they should be interpreted as associative and partially mechanistic rather than as definitive proof of causality.

Maternal diabetes-associated NTDs are likely driven by interacting mechanisms rather than a single pathway. Previous work has implicated oxidative stress, mitochondrial dysfunction, endoplasmic reticulum stress, apoptosis, autophagy dysregulation, miRNA-mediated signaling, and Wnt pathway disruption in diabetic embryopathy (Pacini et al., 2004; Salbaum and Kappen, 2010; Prakasam et al., 2014; Gu et al., 2016; Yu et al., 2016; Wang et al., 2017; Bai et al., 2018). Our findings add BHB-associated epigenetic dysregulation to this framework. Elevated BHB may interact with stress-response pathways because metabolic changes can affect redox balance, chromatin-modifying enzymes, and developmental gene-expression programs (Shi et al., 2014). Therefore, the present model should be viewed as complementary to established diabetic embryopathy mechanisms. Recent work has further shown that targeting inflammatory cell-death pathways can ameliorate maternal diabetes-induced embryonic NTDs in experimental models, supporting the broader concept that mechanism-guided intervention may be feasible (Zhou et al., 2025).

BHB is not only an energy substrate but also a signaling metabolite capable of modulating HDAC activity and histone acetylation (Shimazu et al., 2013; Li et al., 2020). In the present study, diabetic NTD embryos and BHB-treated HT-22 cells showed reduced HDAC1/3 expression and increased H3K27ac. HDAC1/3 overexpression in BHB-treated cells reduced H3K27ac, supporting a regulatory link in this *in vitro* system. However, the *in vivo* developmental sequence remains to be tested directly. Thus, our data indicate that BHB is associated with HDAC1/3-H3K27ac changes under diabetic/NTD-related conditions, while future embryo-based rescue studies are needed to determine whether restoring HDAC1/3 activity prevents NTD phenotypes.

H3K27 acetylation (H3K27ac) is closely associated with transcriptional activation and is a common epigenetic target in cellular regulatory processes (Gates et al., 2017). Studies have shown that treatment of cells with MS-275—an inhibitor of HDAC1 and HDAC3, which, like BHB, selectively targets Class I HDACs—significantly increases H3K27ac levels (Zhang C. et al., 2018). Moreover, our research group previously found that alterations in H3K27 trimethylation affect the expression of downstream HOX genes and contribute to the pathogenesis of neural tube defects (NTDs). This study investigates the role of H3K27ac in maternal diabetes-induced NTDs. Our *in vivo* experiments demonstrated that in the brain tissue of NTD-affected embryos from diabetic mothers, HDAC1 and HDAC3 expression levels were downregulated, whereas H3K27ac levels were elevated (**Figure 2**). Consistently, *in vitro* experiments showed that BHB treatment of HT-22 cells led to decreased expression of HDAC1 and HDAC3 and a concomitant increase in H3K27ac. Furthermore, overexpression of either HDAC1 or HDAC3 in BHB-treated cells effectively reduced H3K27ac levels, providing direct evidence that BHB modulates H3K27ac by suppressing HDAC1 and HDAC3 activity (**Figure 3**).

H3K27ac is commonly enriched at active promoters and enhancers and is associated with transcriptional activation (Wang et al., 2016; Gates et al., 2017). The UCSC Genome Browser prediction suggested H3K27ac enrichment near the Mest promoter, and we observed increased Mest/MEST expression in diabetic NTD embryonic brain tissue. Nevertheless, these data do not directly demonstrate H3K27ac occupancy at the Mest promoter. Chromatin-based assays such as ChIP-qPCR or CUT&Tag are required to confirm direct H3K27ac enrichment and transcriptional regulation at this locus. Similarly, the observed inverse relationship between MEST and mature LRP6 is consistent with previous reports that MEST can affect LRP6 maturation (Jung et al., 2011), but MEST gain- and loss-of-function experiments are required to demonstrate that MEST mediates LRP6 maturation defects in this model.

This study has several limitations. First, direct H3K27ac occupancy at the Mest promoter was not tested by ChIP-qPCR or CUT&Tag, so the proposed H3K27ac-Mest transcriptional link remains inferential. Second, MEST gain- or loss-of-function experiments were not performed; therefore, the causal role of MEST in regulating LRP6 maturation under BHB exposure remains to be established. Third, downstream Wnt/β-catenin pathway activity was not directly measured. Fourth, HT-22 cells are hippocampal neuronal cells and do not reproduce the biology of neuroepithelial cells during neural tube closure. Fifth, additional litter-based and culture-batch-based analyses would further strengthen phenotypic quantification.

Although the present study did not perform *in vivo* BHB-HDAC-H3K27ac intervention experiments, the cell rescue results provide a rationale for future studies testing whether reducing pathological BHB exposure or restoring HDAC1/3-H3K27ac balance during the neurulation window can improve neural tube closure *in vivo*. Such studies should first be performed in ex vivo embryo culture and maternal diabetes animal models, with careful attention to intervention timing, maternal metabolism, placental transfer, and fetal safety. Large-scale human evidence linking maternal diabetes with adverse offspring neurodevelopmental outcomes also highlights the translational importance of prevention-oriented strategies, although direct clinical application will require substantial preclinical validation (Ye et al., 2025).

In summary, our data support the hypothesis that elevated BHB in maternal diabetes is associated with HDAC1/3 suppression, increased H3K27ac, increased MEST expression, and reduced mature LRP6. This pathway may contribute to diabetes-associated NTDs, but direct chromatin validation, MEST functional testing, downstream Wnt/β-catenin analysis, and embryo-based rescue experiments will be necessary to establish causality.

## Data Availability

The raw data supporting the conclusions of this article will be made available by the authors, without undue reservation.
